# Data from Digital Health Devices Informs Ideal Cardiovascular Health

**DOI:** 10.3390/jpm11030189

**Published:** 2021-03-10

**Authors:** Jane A. Leopold, Roger B. Davis, Elliott M. Antman

**Affiliations:** 1 Division of Cardiovascular Medicine, Brigham and Women’s Hospital, Harvard Medical School, 75 Francis Street, Boston, MA 02115, USA; eantman@rics.bwh.harvard.edu; 2 Department of Medicine, Beth Israel Deaconess Hospital, Harvard Medical School, 300 Brookline Avenue, Boston, MA 02215, USA; rdavis@bidmc.harvard.edu

**Keywords:** digital health devices, ideal cardiovascular health, participant-reported outcomes, surveys

## Abstract

Ideal cardiovascular health is associated with a decrease in adverse cardiovascular events. The My Research Legacy study examined ideal cardiovascular health using the Life’s Simple 7 survey and data from digital health devices. We hypothesized that digital devices provide a more objective view of overall cardiovascular health status than self-reported measures. Therefore, we analyzed weight and activity data recorded by digital devices to recalculate the Life’s Simple 7 Health Score. All study participants (*n* = 1561) answered the survey, while a subgroup (*n* = 390) provided data from digital devices. Individuals with digital devices had a lower body mass index (BMI) and higher weekly minutes of vigorous exercise than participants without digital devices (*p* < 0.01). Baseline Health Scores were higher in individuals with digital devices compared to those without (7.0 ± 1.6 vs. 6.6 ± 1.6, *p* < 0.01). Data from digital devices reveal both increases and decreases in measured vs. self-reported BMI (*p* < 0.04) and weekly minutes of moderate and vigorous exercise activity (*p* < 0.01). Using these data, a significant difference was found between the recalculated and the self-reported Life’s Simple 7 Health Score (*p* < 0.05). These findings suggest that incorporation of digital health devices should be considered as part of a precision medicinal approach to assessing ideal cardiovascular health.

## 1. Introduction

In 2010, the American Heart Association (AHA) established a strategic impact goal for 2020 and beyond: improve cardiovascular health by 20% and reduce cardiovascular mortality by 20% [1]. This ideal was renewed in the 2030 impact goal, which addresses ideal cardiovascular health in the context of overall health, well-being, and health equity [2]. To achieve these goals, a panel of experts defined ideal cardiovascular health as a composite of health behaviors, such as normal body weight, sufficient physical activity, healthy diet, and absence of tobacco use, as well as health factors, including normal blood pressure and normal cholesterol and fasting blood glucose levels. These 7 cardiovascular health metrics were incorporated into the online Life’s Simple 7 survey tool [1,3]. Studies have confirmed that ideal cardiovascular health assessed by Life’s Simple 7 is associated with lower rates of subclinical and incident cardiovascular disease and mortality in cross-sectional and prospective studies [4,5,6,7,8,9,10]. Despite this, a recent longitudinal analysis from the Framingham Heart study found that ideal cardiovascular health has declined over the past 20 years in a community-based population [11].

Given the observed deterioration in ideal cardiovascular health, it is not surprising that cardiovascular disease remains the leading cause of death worldwide, with approximately 17.8 million deaths in 2017 [3]. These findings are commensurate with elevated rates of high blood pressure, diabetes, cholesterol and other lipids, and tobacco use in populations worldwide. Physical inactivity and obesity have also emerged as novel risk factors, with the global prevalence of reported to be 27.5% and 12.0%, respectively. Within the United States, rates of physical inactivity are substantially higher (17.3–47.7%), as is the prevalence of obesity, which was 42.4% in 2017–2018 [12,13]. While the age-adjusted death rate per 100,000 population has decreased for heart diseases over the past 20 years, the rate of decline has slowed substantially over the past 5 years, perhaps due to the high burden of cardiovascular disease risk factors [14]. Taken together, these trends underscore the importance of striving for and maintaining ideal cardiovascular health.

Assessing ideal cardiovascular health using Life’s Simple 7 requires participants to self-report outcome measures that may be subject to participant comprehension, accuracy, and recall bias [15]. In the era of widespread adoption of digital health technologies (e.g., wearable or digital health and activity trackers), incorporation of directly measured health-related outcome measures has been suggested as a mechanism to improve data collection in clinical trials [16,17]. It is estimated that more than 80% of the United States population and 50% of individuals worldwide have smartphones, suggesting that this is a feasible approach [16]. In fact, large-scale studies examining 6-min walk distance and cardiovascular health have been completed using digital health technologies [18].

Therefore, we hypothesized that objective data from digital health devices provides a more unbiased view of cardiovascular health status than participant self-reported measures. We tested this hypothesis in a community-based sample from across the United States who enrolled in the My Research Legacy study.

## 2. Materials and Methods

### 2.1. Study Design and Data Acquisition

My Research Legacy was an American Heart Association direct-to-participant study, conducted entirely online (clinicaltrials.gov: NCT02958098). The study collected self-reported data on cardiovascular health and participants were given the option to contribute weight and exercise data recorded by digital health devices. The study was approved by the Advarra Institutional Review Board (approval #31995) and participants provided written informed consent. Participants were eligible to enroll if they were ≥18 years old, lived in the United States, and had internet access. The informed consent process was developed for both computer and smartphone platforms. Participants were required to provide consent for sharing each category of data collected in the study and could withdraw informed consent for any part of the study at any time. At the end of the consent process, participants were instructed to download a PDF version of the signed consent form. The study was open for enrollment from November 2016 to October 2018.

At study entry, consenting participants self-reported baseline demographic information, including age, sex, and race/ethnicity; answered questions to determine if they had a prior history of cardiovascular diseases defined as myocardial infarction, stroke, atrial fibrillation, systolic heart failure, or aortic dissection; and completed Life’s Simple 7 survey questions about exercise, weight, tobacco use, blood pressure, blood cholesterol and fasting glucose levels, and diet. For participants that did not know their blood pressure, cholesterol, or glucose levels at the time of the survey, Life’s Simple 7 imputed a value to calculate the Health Score [1,2]. Participants were considered to have hypertension if they had a systolic blood pressure ≥130 mmHg, a diastolic blood pressure ≥80 mmHg, or if they reported taking antihypertensive medications. Hypercholesterolemia was defined as a total cholesterol level of ≥200 mg/dL or a self-report of taking cholesterol medications.

The sponsor provided a Fitbit Charge 2 device to individuals who did not have a device to register for the study. Participants that consented to share digital data were provided a unique link to Validic (Validic Inc., Durham, NC, USA) to register a device and begin transmitting data. Weight data were obtained from a participant’s own smart scale that was linked by smartphone app to their digital health device and transmitted data to Validic. Exercise and activity data were collected by Validic and parsed into minutes of light, moderate, or vigorous activity. Moderate or vigorous activity exercise data are determined by the digital health device based on metabolic equivalents (METS), with 3 METS defined as the threshold for moderate activity and 6 METS defined as the threshold for vigorous activity [19]. The algorithms by which the digital health devices take raw data and calculate METS are proprietary. The validity of Fitbit’s active minutes has been demonstrated [20,21,22,23]. Exercise data were assessed for 7 consecutive days in order to compare to survey self-reported data. Average daily step count was determined by obtaining the average count of daily steps over 7 consecutive days. Data were collected by Validic in in 24 h increments and downloaded monthly. The participants did not receive direct feedback from the study regarding any aspect of the data. They were, however, able to view their overall Life’s Simple 7 Health Score online when they answered the survey and view their own weight and activity on the digital health devices and associated smartphone apps or online.

### 2.2. Data Access and Security

Demographic and survey data were uploaded directly to Amazon Web Services secure servers via a platform managed by The Broad Institute (Cambridge, MA, USA) and REAN Cloud LLC (Herndon, VA, USA) and deidentified. The Amazon Web Services environment is accredited with a FedRAMP moderate security level. Data from digital health devices were transmitted to Validic over the internet and deidentified. Collected data were parsed into weight, routine, and fitness categories and downloaded to REAN Cloud servers via calls to Validic using the Validic API. Validic is HIPAA compliant and is certified according to the US-EU and US-Swiss Safe Harbor standards to ensure safe transfer of data. Deidentified survey and digital health device data were provided to the American Heart Association by REAN Cloud.

### 2.3. Statistical Analysis

The sample size required to detect a difference in retention rates with 90% power, an alpha value of 0.05, a probability of drop-out of 0.25, and the assumption of an ~50% dropout rate was 1636 participants. Normality of the data was tested using the Shapiro–Wilk test. Comparisons between categorical variables were performed using the chi-square test or Fisher’s exact test. Comparisons between continuous variables were performed using t-tests or paired t-tests. Nonparametric testing was done using the Wilcoxon-rank sum test or Wilcoxon matched pairs signed rank test as applicable. Data are presented as mean ± SD. *p* values < 0.05 are considered statistically significant. Data were analyzed using Stata 15/SE 15.1 (StataCorp LLC, College Station, TX, USA) and Prism 9.0 (GraphPad, San Diego, CA, USA).

## 3. Results

### 3.1. My Research Legacy Study Cohort

A total of 2267 participants provided informed consent for the study. Of these, 694 chose not to participate further and 12 individuals had incomplete data, resulting in a study cohort of 1561 individuals. Of these, 390 individuals agreed to submit data from digital health devices.

First, we compared self-reported responses to the Life’s Simple 7 survey from participants that did not register digital health devices with those that did. There were no significant differences in age, gender, ethnicity, geographic location or prior history of cardiovascular disease between the groups (Table 1). Self-reported data from individuals who registered digital health devices revealed that they were less likely to have diabetes mellitus or use tobacco but had the same prevalence of hypertension and hypercholesterolemia as individuals without digital health devices. Although there were no differences in self-reported systolic, diastolic blood pressure, or total cholesterol levels, participants who registered digital health devices self-reported lower weights (80.8 ± 20.7 vs. 85.4 ± 25.5 kg, *p* < 0.01) resulting in lower BMIs (28.4 ± 6.7 vs. 30.5 ± 8.6 kg/m^2^, *p* < 0.01) (Table 1).

While there was no difference between the groups with respect to self-reported minutes of moderate exercise per week, individuals with digital health devices self-reported more minutes of vigorous exercise per week (81.2 ± 120.3 vs. 62.7 ± 115.3 min/week, *p* < 0.01) (Table 2). There was also no difference between groups with respect to consumption of fruit and vegetables per day, whole grains per day, and fish per week; however, participants with digital health devices drank fewer sugar-sweetened beverages per week than individuals who were not (1.9 ± 2.9 vs. 2.6 ± 3.5 servings/week, *p* < 0.01). Participants in both groups self-reported similar dietary behaviors with respect to avoiding prepackaged foods, eating out, and avoiding additional salt at home (Table 2).

**Figure 1:**
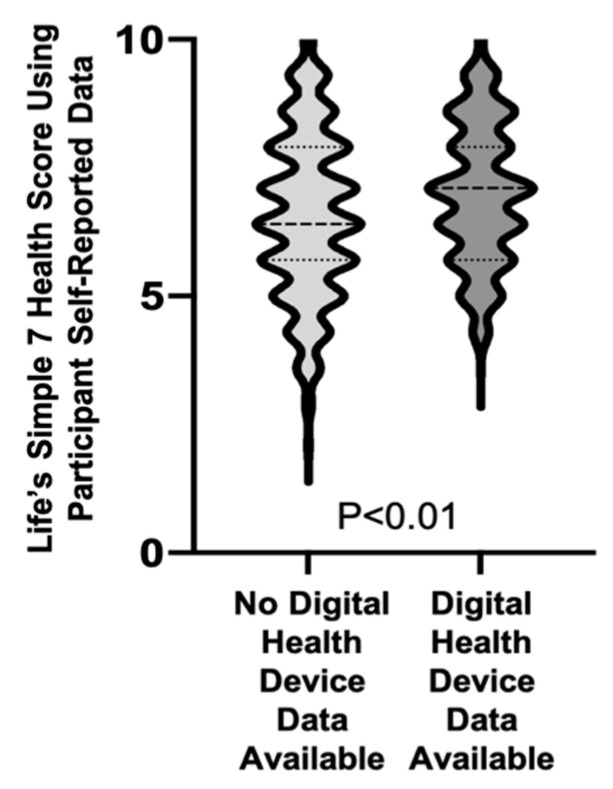
Life’s Simple 7 Health Score calculated using self-reported data for participants with and without registered digital health devices. The distribution of Life’s Simple 7 Health Scores is compared between individuals that did not register digital health devices (*n* = 1171) and those that did register digital health devices (*n* = 390) and presented as violin plots. The median and quartiles are denoted by dashed lines.

When examining the distribution of participants with poor, intermediate, or ideal scores based on self-reported data for each of the Life’s Simple 7 health and lifestyle categories, there were significant differences between the groups with respect to smoking, physical activity, healthy weight, and blood glucose scores. This resulted in participants with registered digital health devices having a significantly better Life’s Simple 7 Health Score based on self-reported data than participants who did not (7.0 ± 1.4 vs. 6.6 ± 1.6, *p* < 0.01) (Figure 1, Table 3).

### 3.2. Incorporating Digital Health Data into Life’s Simple 7 Health Score

Next, we sought to compare data recorded by digital health devices with participant self-reported data. Of the 390 individuals who agreed to contribute digital data, 79.7% registered a Fitbit device, 8.5% registered a Garmin device, 5.9% registered an Under Armour device, and the remainder used other devices or apps. A total of 98 participants did not transmit digital weight data and 35 did not transmit digital exercise data. There were no differences in age, gender, ethnicity, or reported weight between individuals that did or did not transmit digital-health-device-measured weight data. Individuals that did not transmit weight data, however, were more likely to live in the western part of the United States (*p* < 0.01). There were no differences in age, gender, ethnicity, region, or reported moderate exercise minutes per week between individuals that did or did not transmit digital-health-device-measured exercise data. Individuals that did transmit digital data self-reported significantly more weekly minutes of vigorous exercise activity per week than those that did not (87.6 ± 123.7 vs. 16.1 vs. 39.9 min per week, *p* < 0.01).

**Figure 2:**
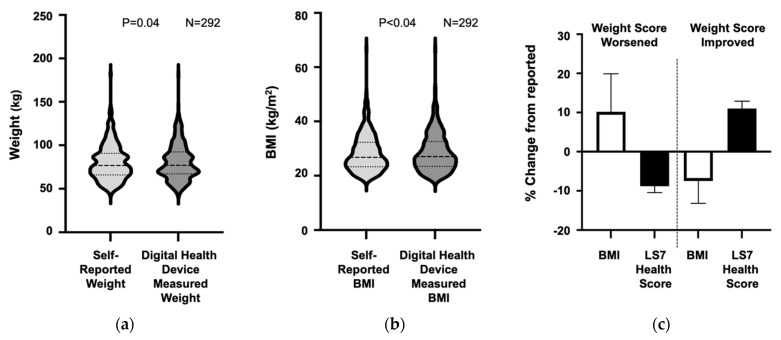
Participant self-reported versus digital-health-device-measured weight and body mass index. Participant self-reported (**a**) weight and (**b**) calculated body mass index (BMI) are compared with weight measured by digital devices and BMI calculated using digital-health-device-measured weight. Data are summarized graphically in violin plots. Median and quartiles are denoted by dashed lines *n* = 292). (**c**) There are both increases and decreases in weight identified by digital health devices within the study population. The effect of a large difference in measured versus reported weight, change in Weight Score, and the resultant effect on Life’s Simple 7 Health Score is illustrated. Digital-device-measured weight was used to calculate body mass index (BMI) and a Life’s Simple 7 Weight Score. In 18 participants, the digital-device-measured BMI was significantly higher than the participant’s self-reported weight and the Weight Score decreased by one class. The digital health device Weight Score was used to calculate the Life’s Simple 7 Health Score. Thirteen participants overestimated their weight (and BMI) resulting in a better Weight Score and overall Life’s Simple 7 Health Score. LS7, Life’s Simple 7.

When comparing self-reported versus digital-health-device-measured weight, participants (*n* = 292) tended to self-report a lower weight (80.4 ± 20.5 vs. 80.9 ± 20.7 kg, *p* = 0.04) and resulting BMI (28.4 ± 6.9 vs. 28.6 ± 7.0 kg/m^2^, *p* < 0.04) than was measured by the device (Figure 2a,b). The average difference between self-reported and measured weight was -1.2 ± 7.9 kg (*p* < 0.04), indicating underreporting of weight. Based on device-measured weight, 31 participants had a reclassification of their Life’s Simple 7 Weight Score, resulting in a significant difference in the distribution of participants with poor, intermediate, and ideal weight scores compared to the Weight Score based on self-reported data (*p* < 0.01). In 18 participants whose Weight Scores worsened using digital health device data, there was a significant decrease in their overall Life’s Simple 7 Health Score (8.3 ± 1.2 vs. 7.6 ± 1.2). In 13 participants whose Weight Scores improved with digital health data, there was a concomitant increase in their Life’s Simple 7 Health Score (6.6 ± 1.1 vs. 7.4 ± 1.1) (Figure 2c).

**Figure 3:**
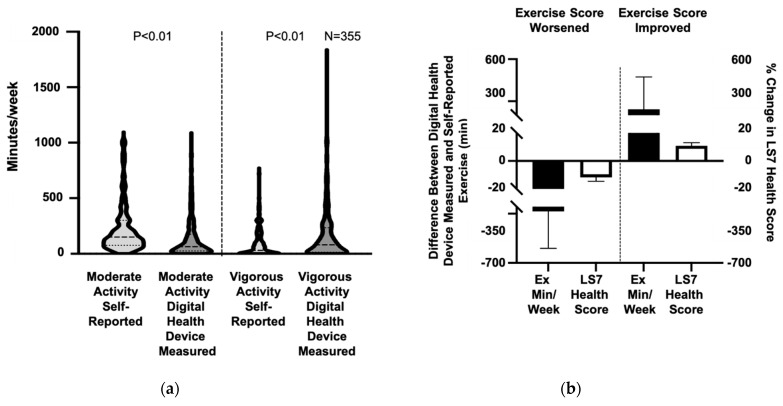
Self-reported exercise activity compared to digital-health-device-measured exercise activity. (**a**) Participant self-reported moderate and vigorous exercise minutes/week were compared with digital-device exercise moderate and vigorous exercise minutes per week and summarized graphically in violin plots. Median and quartiles are denoted by dashed lines, *n* = 355. (**b**) Digital-device-measured exercise (Ex) minutes (Min) per week were calculated. There are both increases and decreases in exercise minutes identified by digital health devices within the study population. The effect of a large difference in measured versus reported exercise, change in Activity Score, and change in Life’s Simple 7 Health Score is illustrated. In 74 participants, digital-device-measured exercise is less than reported exercise minutes and Activity Score and Health Score decreased. In 54 participants, the measured exercise minutes are higher than reported and the Activity Score and Health Score increased. LS7, Life’s Simple 7.

**Figure 4:**
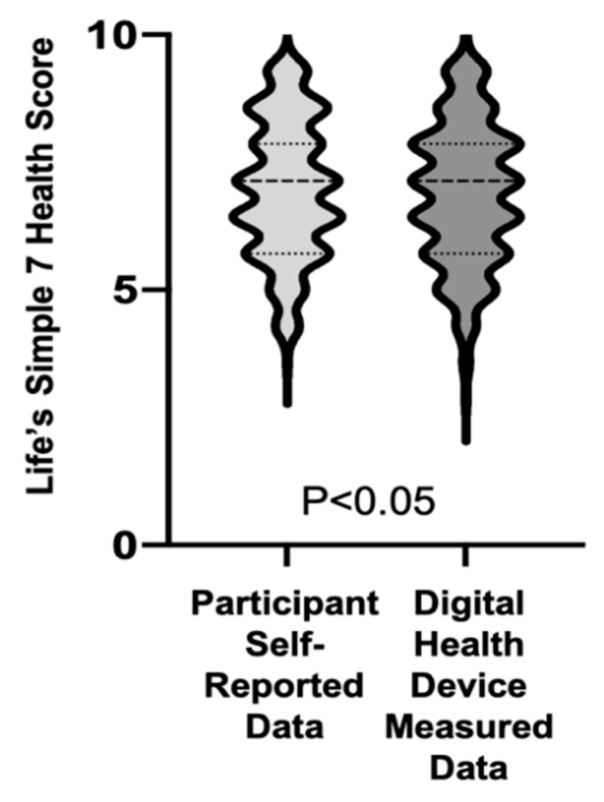
Life’s Simple 7 Health Score based on digital health device data. Life’s Simple 7 Health Score calculated with digital-health-device-measured body mass index and exercise data was compared to the Life’s Simple 7 Health Score calculated with participant self-reported data. Results are summarized graphically in violin plots. Median and quartiles are denoted by dashed lines (*n* = 272).

We next assessed digital device recorded activity data over 7 consecutive days and compared this to participant self-reported data for weekly activity *(n* = 355). Although participants self-reported an average of 215.2 ± 214.0 min of moderate activity weekly, digital health devices recorded an average of 135.3 ± 175.3 min/week, resulting in a mean difference of 79.9 ± 251.9 min/week between self-reported and measured moderate activity (*p* < 0.01) and indicating overreporting of moderate activity. In contrast, digital health devices recorded 164.5 ± 229.3 min of weekly vigorous activity compared with the self-reported 87.6 ± 123.7 min of vigorous activity, a mean difference of −76.9 ± 225.2 min/week between self-reported and measured vigorous activity (*p* < 0.01) (Figure 3a). Using the device-measured activity levels to recalculate the Activity Score, 128 participants had a reclassification of their score resulting in a significant difference in the distribution of participants with poor, intermediate, and ideal activity scores (*p* < 0.01). Among the participants who had their score reclassified, 54 participants had an improvement in their Activity Score classification and Life’s Simple 7 Health Score (6.2 ± 1.3 vs. 6.9 ± 1.3), while 74 had a decline in their score resulting in a decrease in their Life’s Simple 7 Health Score (7.2 ± 1.4 vs. 6.4 ± 1.4) (Figure 3b). For individuals that had both digital-health-device-measured weight and activity data (*n* = 272), incorporating these data into the Life’s Simple 7 Health Score resulted in a decrease in the score compared to using self-reported data (6.9 ± 1.4 vs. 7.0 ± 1.3, *p* < 0.05) (Figure 4).

We also compared participants with digital health device data who were more adherent with ideal cardiovascular health, defined as having a score of ideal in a minimum of 5 Life’s Simple 7 health factors and behaviors categories (*n* = 112), with those that were considered to be nonadherent, defined as having an ideal score in 2 or less categories (*n* = 85). We found that individuals who were less adherent, using this definition, were older (51.4 ± 9.5 vs. 35.9 ± 11.4 years old, *p* < 0.01), were more likely to be men (28.2% vs. 13.4%, *p* < 0.02), and had a prior history of cardiovascular disease (49.4% vs. 25.9%, *p* < 0.01). There were no differences in race, ethnicity, or region between the groups.

Next, we examined average daily step count in participants whose digital health devices transmitted this data *(n* = 351), as daily step counts have been associated with lower all-cause and cardiovascular mortality [24]. The average daily step count for the cohort was 7802 ± 4694 steps. When participants were stratified by step counts (<4000/day, 4000–7999/day, 8000–11,999/day, or ≥ 12,000/day) [24], there was no significant difference between the groups with respect to age, gender, ethnicity, or region. There were, however, significant differences in digital-health-device-measured BMI (*p* < 0.04) and consumption of sugar-sweetened beverages (*p* < 0.05) between the groups with larger BMI and increased consumption of sugar-sweetened beverages was more likely to occur in lower step count groups. There were also significant differences between step count groups with respect to digital-device-measured weekly minutes of moderate or vigorous activity (*p* < 0.01), with lower levels of activity associated with fewer daily steps. Life’s Simple 7 Health Scores using digital heath device data differed between step count groups with higher scores associated with more daily steps (*p* < 0.01).

## 4. Discussion

In the My Research Legacy study cohort, the primary finding was that there are significant differences between participant self-reported and digital-health-device-measured weight and minutes of moderate and vigorous activity per week. Participants both underreported and overreported their weight on the Life’s Simple 7 survey. When compared with digital-health-device-measured weight, these differences were sufficient to change the Weight Score component as well as the Life’s Simple 7 Health Score in 10% of individuals with available weight data. Analysis of digital health device data also revealed that participants tended to under- and overestimate their minutes of exercise activity, resulting in a change in the Activity score and Health Score of 24% of the participants. In addition, we also describe differences in the self-reported burden of cardiovascular disease risk factors, health data, diet, and exercise minutes between participants that registered digital health devices and those that did not. Individuals that did not register devices were less likely to achieve ideal cardiovascular health scores in each of the Life’s Simple 7 health and lifestyle categories.

Prior studies have demonstrated that health measures required to complete the Life’s Simple 7 survey, such as weight, may be underestimated while physical activity is systematically overestimated as compared to digital-health-device-measured weight or activity data [25,26]. Our findings are in line with other studies. One study that included a mixed-community-based population of 2676 adults (mean age 40.7 years) from 18 separate studies found that participants underreported their weight, resulting in misclassifications of obesity for 4% of women and 8% of men [27]. This was confirmed in the Cancer Prevention Study-3. In a subset of 2643 participants, weight was underreported, resulting in 7% of women and 13% of men being misclassified into a lower BMI category [28]. In the aforementioned studies, weight was measured during an in-person study-designated visit, which is in contrast to My Research Legacy where weight was measured by digital health devices in the participant’s home. Although the accuracy of self-reported data should be high when the device used by the participant to measure weight is the same as the digital health device that measures weight, our study shows that discrepancies between self-reported and digital health device weight persist.

In My Research Legacy, we also found differences between self-reported and digital-health-device-measured weekly minutes of exercise. These differences are similar to what was reported in the MyHeartCounts study, where participants were asked to rate their activity level and weekly minutes of self-reported physical activity were compared to the time spent exercising—as recorded by a motion tracker. While there was an association between self-reported activity minutes and perceived activity level, the correlation between the two was small. While the investigators reported questionnaire-based minutes of activity per week as well as digital-device-measured minutes of vigorous activity per week, they did not directly evaluate differences between self-reported and device-measured activity minutes [18].

Our study has several limitations that could influence our findings. First, our study enrolled a predominantly white cohort, so findings may be less broadly applicable to other populations. We also assumed that digital literacy skills were equivalent among individuals who participated in the study; however, it is likely that differences here contributed to the drop-off of participants after providing informed consent. Registration of a digital health device was not mandated as part of the study. This could have biased the digital health device group towards one that is healthier and more physically active. Similarly, the brand or type of digital health device was not prespecified, although the overwhelming majority of participants registered Fitbit or Garmin digital health devices. Differences between digital health devices exist, and studies that compare the accuracy of Fitbit, Garmin, and other digital health devices have been performed [20,29,30,31,32,33]. In general, Fitbit and Garmin devices, when tested head-to-head, demonstrated good heart rate accessibility and small negative bias [29,30]. In addition, we did not collect socioeconomic data and it is possible that underserved populations were not well represented in the study cohort [16]. Although the study did not provide direct feedback to participants, they were able to view their overall Life’s Simple 7 Health Score online when completing the survey as well as their weight and activity data on their devices and associated apps, which may have influenced behavior. In addition, there are many other personality factors, health factors, and behaviors that were not captured by our study that may influence our findings. Finally, we did not prescribe a minimum amount of time that an activity tracker needed to be worn on a daily basis, which could impact the quantity of data recorded. Nonetheless, we were able to determine if the device was worn or used based on data output from Validic.

Remote monitoring of cardiovascular health metrics already occurs with devices that record exercise activity, blood pressure and heart rate, weight, oxygen saturation, and electrocardiograms [17]. Future digital-health-device-enabled trials could measure these and other health or lifestyle metrics and have the potential to incorporate recorded data into the electronic health record [16,34]. Digital-health-device-enabled studies also offer the advantage of facilitated informed consent, continuous or near-continuous monitoring, and assessment of novel endpoints with clinical implications, such as step counts. For example, daily step counts measured by digital activity trackers have been inversely associated with type 2 diabetes mellitus outcomes, cardiovascular events, and all-cause mortality [25,35,36].

There are several hurdles that investigators will have to overcome when considering the addition of digital health devices to clinical studies. The first is related to adherence: among individuals with established cardiovascular disease, adherence to the use of digital health devices for exercise or activity monitoring ranged from 39.6% to 85.7% across 10 studies [37]. Second, there is a steep drop-off of participants after they engage with a study. For example, in MyHeartCounts, of the 48,968 participants who provided informed consent, only 9.3% of individuals completed the 7 days of activity monitoring in the study [18]. Third, participants’ attitudes toward the use of digital health devices may be another factor for consideration, as individuals tend to express concern with respect to privacy and surveillance, cost, and usability and understanding [38].

The concept of including digital health devices in clinical studies is attractive as it allows for the inclusion of unbiased objective data into the study. My Research Legacy illustrates the value of these data by demonstrating how it affects a validated score of ideal cardiovascular health. The study also underscores the need for the development or refinement of a survey tool to assess ideal cardiovascular health that incorporates language-specific features for studies with digital health devices. Thus, our findings suggest that data from digital health devices should be included as a precision medicinal approach to provide a dynamic assessment of ideal cardiovascular health.

## Figures and Tables

**Table 1:** Participant self-reported demographics, cardiovascular risk factors, and health factors.

	Entire Cohort (*n* = 1561)	No Digital Health Device Data Available (*n* = 1171)	Digital Health Device Data Available (*n* = 390)	*p* Value
Age (year)	44.2 ± 13.0	44.5 ± 13.1	43.2 ± 12.7	0.08
Gender (% Female)	80.1	80.6	78.7	0.42
Race and Ethnicity (no.)				
Asian Black Hispanic White Other	42 60 68 1337 54	31 49 49 998 44	11 11 19 339 10	0.55
Region (no.)				
Northeast South Midwest West	227 622 378 334	168 481 269 253	59 141 109 81	0.17
Cardiovascular Disease (%)	36.3	36.5	35.6	0.77
Diabetes mellitus (%)	10.3	11.7	6.2	<0.01
Hypertension (%)	49.8	51.0	46.4	0.12
Hypercholesterolemia (%)	53.4	54.5	50.3	0.15
Medications (%)				
Diabetes mellitus Hypertension Hypercholesterolemia	8.8 32.5 20.6	9.9 34.8 21.2	5.4 25.6 18.7	<0.01 <0.01 0.30
Smoking status (%)				
Current Quit < 12 months Quit ≥ 12 months Never	6.9 3.8 23.6 65.7	8.7 4.2 23.3 63.8	1.5 2.6 24.6 71.3	<0.01
Weight (kg)	84.2 ± 24.4	85.4 ± 25.4	80.8 ± 20.7	<0.01
Height (cm)	167.6 ± 9.6	167.3 ± 9.8	168.4 ± 9.1	0.06
BMI (kg/m^2^)	29.9 ± 8.2	30.5 ± 8.6	28.4 ± 6.7	<0.01
Systolic blood pressure (mmHg) *	118.1 ± 12.7	118.4 ± 13.1	117.1 ± 11.7	<0.07
Diastolic blood pressure (mmHg) *	73.4 ± 8.7	73.5 ± 8.9	72.8 ± 7.8	0.16
Total cholesterol (mg/dL) *	188.7 ± 29.2	189.3 ± 28.6	186.9 ± 31.1	0.17

* Includes imputed data from Life’s Simple.

**Table 2:** Participant self-reported diet and exercise data.

	Entire Cohort (*n* = 1561)	No Digital Health Device Data Available (*n* = 1171)	Digital Health Device Data Available (*n* = 390)	*p* Value
DIET				
Vegetables/day (cups)	1.9 ± 1.3	1.9 ± 1.3	1.9 ± 1.3	0.91
Fruit/day (cups)	1.4 ± 1.1	1.4 ± 1.1	1.3 ± 1.0	0.67
Fish (servings/week)	0.9 ± 1.0	0.9 ± 1.0	0.9 ± 1.1	0.86
Whole grains (servings/day)	1.6 ± 1.2	1.6 ± 1.2	1.7 ± 1.3	0.21
Sugar-sweetened beverages (servings/week)	2.4 ± 3.4	2.6 ± 3.5	1.9 ± 2.9	<0.01
Avoid prepackaged foods (%)	52.2	52.4	51.5	0.76
Avoid eating out (%)	37.6	38.3	35.4	0.30
Avoid salt at home (%)	56.6	56.3	57.7	0.63
EXERCISE				
Moderate exercise (min/week)	204.0 ± 215.0	201.2 ± 216.1	212.2 ± 211.6	0.38
Vigorous exercise (min/week)	67.3 ± 116.8	62.7 ± 115.3	81.2 ± 120.3	<0.01

**Table 3:** Life’s Simple 7 Health Score calculated from participant self-reported data.

	Entire Cohort (*n* = 1561)	No Digital Health Device Data Available (*n* = 1171)	Digital Health Device Data Available (*n* = 390)	*p* Value
Smoking score (%)				
Poor Intermediate Ideal	6.9 3.8 89.4	8.7 4.2 87.1	1.5 2.6 95.9	<0.01
Physical activity score (%)				
Poor Intermediate Ideal	1.9 37.7 60.4	2.2 39.0 58.8	1.0 33.6 65.4	<0.02
Healthy diet score (%)				
Poor Intermediate Ideal	44.2 47.1 8.7	45.0 46.5 8.5	41.8 50.2 9.2	0.28
Healthy weight score (%)				
Poor Intermediate Ideal	42.3 24.2 33.5	44.6 23.0 32.4	35.4 28.0 36.6	<0.01
Blood glucose score (%)				
Poor Intermediate Ideal	3.8 35.8 60.4	4.3 37.0 58.7	2.6 31.8 65.6	<0.02
Cholesterol score (%)				
Poor Intermediate Ideal	2.6 49.8 47.6	2.7 50.8 46.5	2.1 46.9 51.0	0.10
Blood pressure score (%)				
Poor Intermediate Ideal	6.2 52.5 41.3	7.1 52.4 40.5	3.3 52.8 43.9	0.07
Life’s Simple 7				
Health Score	6.7 ± 1.5	6.6 ± 1.6	7.0 ± 1.4	<0.01

## Data Availability

For information regarding data availability, please contact the corresponding author. The data are not publicly available due to privacy restrictions and protection of personal data.
